# Dr. Edward W. Roos

**Published:** 1916-04

**Authors:** 


**Dr. Edward W. Roos,** Buffalo, 1903, was born in Buffalo March 29, 1880. After graduation from the Masten Park High School, he entered the Medical Dept. of the University of Buffalo. After receiving his medical doctorate, he practiced in Howard and Avoca, took a post graduate course in London in 1913, and located in Corning. His health failed and he died in New York city Feb. 26, the funeral being held from his former home in Buffalo.

